# Effectiveness of strengthened stimulation during acupuncture for the treatment of allergic rhinitis: study protocol for a randomized controlled trial

**DOI:** 10.1186/1745-6215-15-301

**Published:** 2014-07-24

**Authors:** Qing Chen, Qinxiu Zhang, Luyun Jiang, Xinrong Li, Yang Liu, Yan Xie, Shan Mu, Ying Liu, Xiaopei Wang, Yunzhi Yu, Min Li

**Affiliations:** Chengdu University of Traditional Chinese Medicine, No.37 Twelve Bridge Road, Chengdu, Sichuan Province 610072 China; Department of Otorhinolaryngology, Head and Neck Surgery of the Teaching Hospital of Chengdu University of Traditional Chinese Medicine, No.39 Twelve Bridge Road, Chengdu, Sichuan Province 610072 China

**Keywords:** Allergic rhinitis, *De qi*, Randomized controlled trial

## Abstract

**Background:**

The traditional Chinese theory of acupuncture emphasizes that the intensity of acupuncture must reach a threshold to generate *de qi* (a specific and compound sensation during the acupuncture), which is necessary to achieve the best therapeutic effect. However, the notion that *de qi* must be achieved for maximum benefit has not been confirmed by modern scientific evidence. This study aims to compare the efficacy of acupuncture with either strong (intended to elicit *de qi*) or weak stimulation among patients with allergic rhinitis.

**Methods/Design:**

This study compares real versus sham acupuncture in 140 patients with a history of persistent allergic rhinitis (PER) or intermittent allergic rhinitis (IAR) and with a positive skin prick test (SPT). The trial will be conducted in the Teaching Hospital of Chengdu University of Traditional Chinese Medicine (China). In the study, patients will be randomly assigned into two groups by computer-generated randomization and assessed prior to treatment. They will then receive 12 sessions of treatments for 4 consecutive weeks and have a follow-up phase lasting 12 weeks. The main outcome measures include the primary and secondary indicators. Primary indicators are subjective symptoms scores as evaluated by visual analogue scales (VAS), rhinoconjunctivitis quality of life questionnaires (RQLQ), and the Modified Massachusetts General Hospital acupuncture sensation scale, Chinese version (C-MMASS). The secondary indicators are the results of laboratory examinations, such as serum allergen-specific immunoglobulin E (sIgE) nasal inflammatory cells counts (mast cells, eosinophils, and T cells), and nitric oxide concentration in nasal excretion. The use of anti-allergic medication will also be recorded as one of the secondary indicators. Furthermore, adverse events will be recorded and analyzed. Intention-to-treat analysis (ITT) and per-protocol (PP) analysis will be performed to test and verify the results in this trial.

**Discussion:**

The results of this trial will demonstrate the efficacy of using acupuncture to treat allergic rhinitis and verify whether the effectiveness of acupuncture is related to the needle sensation *de qi*.

**Trial registration:**

Chinese Clinical Trial Registry: ChiCTR-TRC-13003594 (registered on 16 August 2013, and the first patient was randomized on 27 September 2013).

**Electronic supplementary material:**

The online version of this article (doi:10.1186/1745-6215-15-301) contains supplementary material, which is available to authorized users.

## Background

Allergic rhinitis (AR) is a symptomatic disorder of the nose resulting from an immunoglobulin E (IgE) -mediated immunological reaction to allergen exposure. Its major symptoms include rhinorrhea, nasal itching, obstruction, and sneezing, all of which are reversible either spontaneously or with treatment [1]. AR has become a major health problem worldwide. It has been estimated by the ARIA (Allergic Rhinitis and its Impact on Asthma) 2008 document that over 600 million patients from all countries, in all ethnic groups, and of all ages suffer from AR [2]. It dramatically impacts quality of life (QOL) and creates a high economic burden. The conventional treatment of AR symptoms, such as nasal obstruction, rhinorrhea, sneezing, and itching, includes the use of intranasal corticosteroids, oral antihistamines with or without decongestants, immunotherapy, and education [3]. A remarkable number of patients with AR are not satisfied with conventional medical treatment for they repeatedly experience side effects and incur additional healthcare costs. As an available and affordable treatment choice, acupuncture has been accepted as a therapeutic option for relieving symptoms of AR in China and other countries [4–6].

However, studies on the efficacy of acupuncture in patients with AR have yielded inconclusive results. For example, Choi *et al*. [7] stated that active acupuncture showed a significantly greater effect on symptoms of allergic rhinitis than sham acupuncture, while Magnusson *et al*. [8] found that there were no differences in clinical symptoms which had been seen between active versus sham acupuncture. At the same time, a number of trials have the same situation in migraine [9, 10], low back pain [11, 12] and some other diseases. All of those trials did not find verum acupuncture more effective than sham acupuncture and did not emphasize that the verum acupuncture group must have the needle sensation of *de qi*.

*De qi* is an internal compound sensation of soreness, tingling, fullness, aching, coolness, warmth, heaviness, and a radiating sensation at and around acupoints, and is elicited by manipulation of the needles (rotated as well as being moved both upward and downward). According to the traditional theory and clinical practice, *de qi* is one of the fundamental characteristics of acupuncture. The importance of *de qi* was first mentioned *circa* 100 BC, in the Huangdi Neijing (The Yellow Emperor’s Classic of Internal Medicine). For example, Ling Shu (chapter 1) [13] reads, ‘For acupuncture to be successful, the qi must arrive (qi zhi). Acupuncture’s effects come about like the clouds blown away by the wind.’ Another example is in Ling Shu (chapter 9) where it is advised that ‘The acupuncturist should devote all his/her concentration to the needle, keep the needle on the surface and move it gently, until the qi has arrived (qi zhi).’ There is a long-held belief in the theory of acupuncture that the intensity of the stimulus must reach a certain threshold to elicit *de qi*, which plays a pivotal role in achieving the best therapeutic effects [14]. So far, more and more studies have begun to assess *de qi* by using different scales, and to investigate the relationship between *de qi* and the therapeutic effects [15, 16]. However, this long-held belief has not been confirmed by sufficient evidence from randomized controlled trials about AR.

As mentioned above, we have designed a randomized, double blind, large scale clinical trial to compare the efficacy of acupuncture with either strong (intended to elicit *de qi*) or weak stimulation among patients with AR. The design and methodologies of this study have been approved by the Sichuan Regional Ethics Review Committee on Traditional Chinese Medicine with an ethics approval number of 2013KL-025. The work reported in this article is registered with the Chinese Clinical Trial Registry (registration number: ChiCTR-TRC-13003594).

## Methods/Design

### Study design

**Figure 1:**
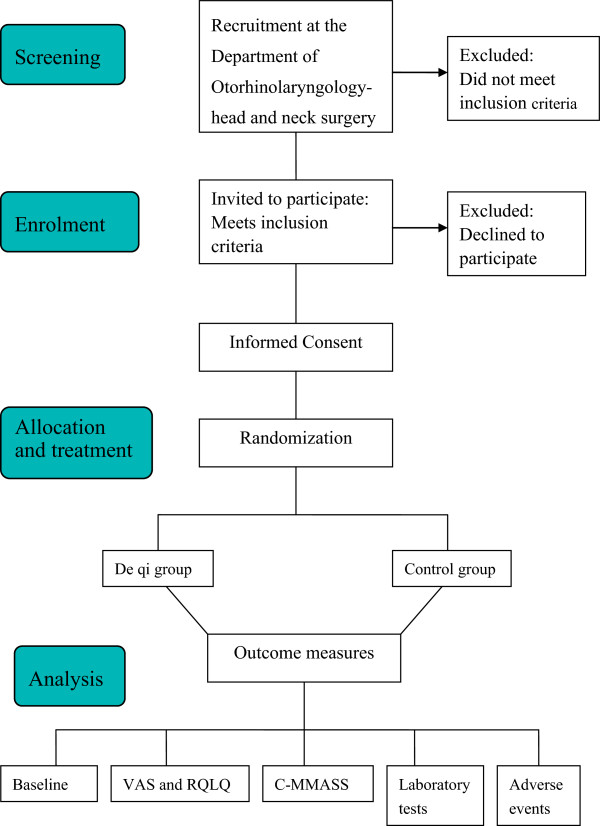
**Screening, recruitment, randomization, and participant follow-up schedule of the trial.** C-MMASS, Modified Chinese version of the Massachusetts General Hospital Acupuncture Sensation Scale; RQLQ, Rhinoconjunctivitis Quality of Life Questionnaires; VAS, Visual analog scales.

This trial is a single center, double blind, sham group randomized controlled trial, which will be performed at the Department of Otorhinolaryngology, Head and Neck Surgery, Teaching Hospital of Chengdu University of Traditional Chinese Medicine, China. The study will be sequentially conducted as follows: a run-in period of 2 weeks prior to randomization, a treatment period of 4 weeks (3 sessions per week), and a follow-up period of 12 weeks. The total study period will be 18 weeks. At the end of the run-in period participants will be randomized to the active acupuncture group or the sham acupuncture group by a computer-generated random number list (Figure 1).

### Randomization and blinding

The randomization sequence will be computer generated by independent research staff using software called Statistical Program for Social Sciences (SPSS 18.0 IBM China Company headquarters, Beijing, China). After randomization, group assignments will be concealed in light-proof and sealed sequentially numbered envelopes. The included participants will be randomly enrolled by sealed envelope and assigned to the *de qi* group or sham-acupuncture control group in a ratio of 1:1. Then the person in charge of the envelopes will give the specified envelope to the acupuncturist. To preserve masking, only the acupuncturists will have access to the treatment allocation.

Randomization will be performed by researchers who are not in direct contact with the participants. Participants, researchers, and study physicians who interview and recruit patients are blinded to the group assignments. The acupuncture practitioners will be informed due to the nature of the intervention, but they will be asked not to communicate with the participants or assessors regarding treatment procedures and responses.

The two randomized groups will be named A or B in the medical records and the case report forms will not be directly linked to the treatment. All information will be locked out and saved in a database before statistical analysis is performed. After statistical analysis, the blind exposure will be provided.

### Inclusion criteria

Each participant must have a diagnosis of AR by an Otolaryngology doctor, according to the criteria listed in ‘Allergic Rhinitis and its Impact on Asthma’ (ARIA 2008 [2]) Patients who will be recruited in this study should meet the following inclusion criteria: (1) Male or female aged between 18 and 65 years. (2) Presenting with typical symptoms of AR, such as rhinorrhea, sneezing, nasal obstruction, and pruritus. These symptoms should last for more than one hour on most days. Some patients may have ocular symptoms due to outdoor allergens. (3) Having at least one positive result among the following laboratory findings: elevated total blood IgE level or positive skin prick test reaction. (4) Providing an informed written approval. (5) No current participation in any other clinical trials.

### Exclusion criteria

Patients with any of the following conditions will be excluded: (1) Patients are receiving immune therapy. (2) Patients have received systemically administered corticosteroids, antihistamines, or decongestants within the most recent six months. (3) Patients with other allergic diseases, such as bronchial asthma or allergic purpura. (4) Patients with nasal polyposis and congenital nasal abnormalities including nasal dermoid cysts and congenital midline nasal masses, sinusitis, or asthma. (5) Patients with serious medical conditions, such as liver or kidney dysfunction, severe dyslipidemia, AIDS, vascular malformation, hypertension, hematologic diseases, diabetes mellitus, a past or current malignant tumor, mental disorders and other infectious or systemic diseases that would make treatment with acupuncture inappropriate.(6) Pregnant women or women who have been trying to get pregnant in the last six months, or women who are lactating.

### Withdrawal from the study

All participation in the trial is voluntary. Participants have the right to withdraw from the study at any time for any reason without any consequences for further medical treatment. Also, investigators have the right to terminate the participation of any patients if any severe adverse events occur or any events that may hurt the participant’s interest. The reasons and circumstances for study discontinuation will be documented in the case report form. All data are analyzed based on the intention-to-treat (ITT) principle.

### Recruitment

Patients will be recruited at the otorhinolaryngology clinic at the Affiliated Hospital of Chengdu University of Traditional Chinese Medicine. At the same time, television advertisements will be broadcast on local channels to promote study participation. Advertisements will also be published in local newspapers. Printed recruitment posters will be posted in the hospital.

### Interventions

During the treatment process, all participants will be explained the meaning of acupuncture and *de qi* by the acupuncturists in the same way. Both the *de qi* and control group participants will receive acupuncture treatments respectively, 3 times a week for a total of 12 sessions over 4 weeks. The same number and type of needles (Hwato disposable sterile acupuncture needles Suzhou Medical Supplies Co., Ltd., Suzhou, China) will be used for both groups. The needle selected was 0.30 mm in diameter and the length of the needle selected varied according to the point location. Needle sites in both active and sham acupuncture groups will be swabbed with 75% alcohol before insertion. On withdrawal of the needle, dry sterilized cotton balls will be firmly applied to the insertion points. Medications which could affect the allergic rhinitis symptoms are not permitted.

In the *de qi* group, the needle was inserted transversely, obliquely, or perpendicularly depending on the acupoints selected with 10 to 30 mm in depth. The acupuncturist will manipulate the needles manually after insertion, using techniques such as lifting, thrusting, and twirling, until *de qi* was achieved. The needles will be left in place for 30 minutes. During the treatment session, the patient will be asked about *de qi* every 10 minutes and the needles will be manipulated to maintain the intensity of the sensation (Table 1). In the control group the needles will be inserted to a depth of 3 to 5 mm perpendicularly to the skin and left in place for 30 minutes without manipulation. Patients in the other treatment group are treated in different rooms. All sessions for each patient were performed by the same acupuncturist.

**Table 1:** **Needling procedure for** ***de qi*** **group**

Real point	Angle and Direction	Depth (mm)
LI20 (both)	Obliquely along the nasolabial sulcus towards the root of the nose with respect to the skin	10-15
Yingxiang		
LI4 (both)	Perpendicular to the skin	20-30
Hegu		
LI11 (both)	Perpendicular to the skin	20-30
Quchi		
ST36 (both)	Perpendicular to the skin, between the tibia and the fibula	20-30
Zusanli		
EX-NH3 (unilateral)	Transversely, downward towards the root of the nose	10-20
Yingtang		

The acupuncture intervention was based on traditional Chinese acupuncture theory and previous Chinese literature. These acupoints (bilateral: Yingxiang-LI20, Hegu-LI4, Quchi-LI11, Zusanli-ST36, unilateral: Yintang -EX-HN3) are selected according to the ‘WHO Standard Acupuncture Point Location’ [17]. According to traditional acupuncture theories, we know that the key pathogenesis of AR is an invasion of pathogenic agents such as cold, wind, and damp, which lead to the imbalance of the large intestine, stomach meridian, or governor vessel. Thus, the most frequently used specific acupoints for these meridians would be selected in this trial according to a previous review of ancient and modern literature. Yingxiang (LI20) is considered as a specific point on Yangming’s large intestine meridian of hand. Hegu (LI4) is the source point of the large intestine meridian. Therefore, LI20, ST36, and EX-NH3 are selected to disperse pathologic *Qi* and blood from these meridians and improve the function of the nose and sinuses. The ancient Chinese believed that Yangming is a meridian abundant with blood and *Qi* and is the acquired foundation. Zusanli and Quchi will be used to fortify the spleen and replenish lung *Qi*.

### Ethics

This trial is performed according to the principles of the Declaration of Helsinki (Edinburgh 2000). The trial protocol has been approved by the Sichuan Regional Ethics Review Committee on Traditional Chinese Medicine. Written informed consent will be obtained from each participant before registration. All patients will be given enough time to decide whether they would sign the informed consent, or they will be given treatment options other than acupuncture if they are not willing to participate.

### Outcome measurements

#### Primary outcomes

For the evaluation of the main outcome measures patients are requested to fill in a questionnaire including the Rhinitis Quality of Life Questionnaire (RQLQ) [18, 19], the visual analogue scale (VAS) [20] and C-MMASS (Massachusetts General Hospital Acupuncture Sensation Scale, modified Chinese version) [21].

In VAS, the severity of the combination of the nasal symptoms will be subjectively rated by the study patients on the scale. The severity of symptoms can be divided into mild, moderate, or severe based on the total severity VAS score (0 to 10 cm: mild, VAS 0 to 3; moderate, VAS 3.1 to 7; severe, VAS 7.1 to 10). VAS scores will be recorded every two weeks throughout the whole trial.

The RQLQ (see Additional file 1) evaluates impairment of everyday life (activity, sleep, everyday life problems, condition of health) caused by symptoms of the eyes and nose. It contains 28 questions related to these dimensions. Each question can be divided into seven degrees: 0 - not trouble, 1 - any trouble at all, 2 - somewhat troubled, 3 - moderately troubled, 4 - quite a bit of trouble, 5 - very troubled, 6 - extremely troubled. It will be measured by questionnaire at baseline, at the end of treatment, during the follow-up period, and at the end of the follow-up period (a total of five times). Both the scores of VAS and RQLQ questionnaire will be blindly assessed.

C-MMASS (see Additional file 2) is an accepted method of objectively scoring the sensation of *de qi*. Immediately after removing the needles during the 1st, 3rd, 5th, 7th, 9^th^, and 12^th^ sessions, patients will be asked to fill in the questionnaire. Patients were questioned about whether each of the sensations of *de qi* had occurred during the session.

### Secondary outcomes

Secondary outcome measures include serum allergen-specific IgE, blood eosinophil count, nasal inflammatory cells counts (mast cells, eosinophils, and T cells), the level of nitric oxide concentration in nasal secretion, and side effects. The patients were examined by the otolaryngologist at the following times: on entering the study, at the end of the run-in period, in the middle and at the end of the treatment phase, and every 4 weeks during the follow-up phase. Nasal secretions and blood samples will be collected on entry, at the end of the treatment phase, and at 4 and 8 weeks after the end of treatment. Adverse events of acupuncture, if any, will be checked and recorded (Table 2).

**Table 2:** **Outcome measurements**

Week	-2	0	1	2	3	4	6	8	10	12	14	16
	Baseline		Treatment phase 12 sessions				Follow-up phase					
Patient enrollment	√											
Medical history	√											
Sign the informed consent		√										
Skin prick tests		√										
Randomization		√										
VAS		√		√		√	√	√	√	√	√	√
RQLQ		√				√		√		√		√
C-MMASS			√	√	√	√						
Laboratory		√				√		√				√
Adverse event		√	√	√	√	√	√	√	√	√	√	√

C-MMASS, Modified Chinese version of the Massachusetts General Hospital Acupuncture Sensation Scale; RQLQ, Rhinoconjunctivitis Quality of Life Questionnaires; VAS, Visual analog scales. The numbers at top of figure are weeks. On the left of the figure, it shows weeks.

To test the effectiveness of blinding, patients in the acupuncture and sham acupuncture groups fill in a questionnaire after the third acupuncture session to assess the credibility of the respective treatment methods [22].

### Sample size calculation

Considering participants recruited in the two groups are in a 1:1 ratio, the following formula was used to estimate sample size [23]:
1

In the formula: n1, n2 represent the sample size of each group. π_1_, π_2_ is the overall rate of each sample, respectively. π_c_ = (π_1_ + π_2_)/2, α = 0.05, Z_0.05_ = 1.96, β = 0.10, Z_0.10_ = 1.282. According to the previous literature [24], statistical analysis will be performed using a 5% significance and 90% power, resulting in an estimated 70 patients per group with the dropout rate of 20%.

### Statistical analysis

Analysis of all data in this trial will be performed by the National Clinical Trial Center of Chinese Medicine (Chengdu GCP Center) in China, which is performed in a blinded manner because analysis of data is done by a special analysis-researcher. The data will be analyzed with SPSS 18.0 and SAS 9.0 (SAS Institute Inc. 100 SAS Campus Drive Cary, NC 27513-2414, USA) statistical software packages. Results will be summarized as means, standard deviations, and 95% confidence intervals (CIs) for continuous data, medians, quartiles, and ranges for rank data, and frequencies and percentages for discrete data.

The first step of analysis is to check whether *de qi* groups were more efficacious in treating AR than the control group. If *de qi* treatment is more effective than the control group in treating AR, the next step is to make comparisons among *de qi* group in the relationship between the effectiveness of treating AR and the scores of the C-MMASS.

The intention-to-treat (ITT) group is defined as the patients who are randomized. The per-protocol (PP) group is defined as the patients who completed the study and do not have major protocol violations. All analyses were based on the ITT group and PP group. And the result of the ITT analysis will be compared with that of the PP analysis to check whether the results are consistent.

## Discussion

The main objective of this trial is to investigate the relationship between the effect of the acupuncture for patients with AR and the needle sensation of *de qi*. This objective has two aspects: one is whether acupuncture with *de qi* is more efficient than without *de qi*; the other is the relationship between *de qi* and efficiency. In this trial, we assess the strength of *de qi* by the score of C-MMASS and the efficiency by the score of RQLQ. We want to know whether a high score equates to high efficiency or not. The result of this trial is expected to provide convincing evidence that strengthened stimulation during acupuncture for the treatment of allergic rhinitis is more effective.

In the literature, sham acupuncture is considered to be a placebo treatment in acupuncture studies. There are two common types of sham control for the clinical study for acupuncture, one is a technique in which needles are inserted shallowly at defined acupoints, another is at locations of 1 to 2 cm away from the defined acupoints. In our study we chose to use the point superficial needle-insertion method. The needles are inserted into the sham points to a depth of 3 mm and are retained at the subcutaneous level. To ensure the efficacy and safety of the treatment, acupuncturists should undergo special training in Teaching Hospital of Chengdu University of Traditional Chinese Medicine and have sufficient knowledge, special technique, and good experience in acupuncture.

## Trial status

This trial is currently recruiting participants (began at 1 September 2013).

## Electronic supplementary material

Additional file 1: **Chinese vision of RQLQ.** Questionnaire to measure the quality of Life. (DOCX 71 KB)

Additional file 2: **C-MMASS.** Questionnaire to objectively scoring the sensation of *de qi*. (DOC 139 KB)

## Abbreviations

AIDS: Acquired Immune Deficiency Syndrome
AR: Allergic rhinitis
C-MMASS: Modified Chinese version of the Massachusetts General Hospital Acupuncture Sensation Scale
IAR: Intermittent allergic rhinitis
ITT: Intention-to-treat
PER: Persistent allergic rhinitis
PP: Pre-protocol
RQLQ: Rhinoconjunctivitis Quality of Life Questionnaires
SPT: Skin prick test
TCM: Traditional Chinese Medicine
VAS: Visual analog scales.
