# Supplementary material for: Effectiveness of strengthened stimulation during acupuncture for the treatment of allergic rhinitis: study protocol for a randomized controlled trial
Source: Trials. 2014 Jul 24;15:301. doi: 10.1186/1745-6215-15-301 (PMC4133069; doi:10.1186/1745-6215-15-301)
Supplement: Supplementary file 1 — Additional file 1: Chinese vision of RQLQ. Questionnaire to measure the quality of Life. (DOCX 71 KB) [file 13063_2013_2188_MOESM1_ESM.docx]

**变应性鼻炎生存质量调查问卷**

填写日期：_______年______月_______日

就您所知，1.您是否对某些物质过敏？若是，请指出过敏物：____________________

2.您家里人或亲戚有过敏性疾病的病史吗？_______________

请填完每一个选项,勿缺漏,谢谢!

**活动**

在过去7天内，该项活动在多大程度上受您的鼻／眼睛症状所困扰

|  | 没有困扰 | 几乎没有困扰 | 有些困扰 | 中等程度困扰 | 十分困扰 | 很困扰 | 极度困扰 |
| --- | --- | --- | --- | --- | --- | --- | --- |
| 1.日常生活工作中的活动（您的工作或您在家必须经常做的事） | 0 | 1 | 2 | 3 | 4 | 5 | 6 |
| 2.社交活动（例如：与家人和朋友的活动，与孩子或宠物玩耍，性生活，爱好等） | 0 | 1 | 2 | 3 | 4 | 5 | 6 |
| 3.户外活动（例如：园艺、晨练、运动、户外静坐等） | 0 | 1 | 2 | 3 | 4 | 5 | 6 |

**睡眠**

在过去的7天,您在多大程度上因您的鼻/眼症状而被以下睡眠问题所困扰?

|  | 没有困扰 | 几乎没有困扰 | 有些困扰 | 中等程度困扰 | 十分困扰 | 很困扰 | 极度困扰 |
| --- | --- | --- | --- | --- | --- | --- | --- |
| 4.入睡困难 | 0 | 1 | 2 | 3 | 4 | 5 | 6 |
| 5.夜间醒来 | 0 | 1 | 2 | 3 | 4 | 5 | 6 |
| 6.夜间睡眠欠佳 | 0 | 1 | 2 | 3 | 4 | 5 | 6 |

**非鼻／眼症状**

在过去的7天里,您在多大程度上因您的鼻/眼症状而被下列问题所困扰?

|  | 没有困扰 | 几乎没有困扰 | 有些困扰 | 中等程度困扰 | 十分困扰 | 很困扰 | 极度困扰 |
| --- | --- | --- | --- | --- | --- | --- | --- |
| 7.精力不足 | 0 | 1 | 2 | 3 | 4 | 5 | 6 |
| 8.口渴 | 0 | 1 | 2 | 3 | 4 | 5 | 6 |
| 9.工作能力下降 | 0 | 1 | 2 | 3 | 4 | 5 | 6 |
| 10.疲倦 | 0 | 1 | 2 | 3 | 4 | 5 | 6 |
| 11.注意力难以集中 | 0 | 1 | 2 | 3 | 4 | 5 | 6 |
| 12.头疼 | 0 | 1 | 2 | 3 | 4 | 5 | 6 |
| 13.疲惫不堪 | 0 | 1 | 2 | 3 | 4 | 5 | 6 |

**实际问题**

在过去的7天里,您在多大程度上因您的鼻/眼症状而被下列问题所困扰?

|  | 没有困扰 | 几乎没有困扰 | 有些困扰 | 中等程度困扰 | 十分困扰 | 很困扰 | 极度困扰 |
| --- | --- | --- | --- | --- | --- | --- | --- |
| 14.因为不得不携带卫生纸而感到不便 | 0 | 1 | 2 | 3 | 4 | 5 | 6 |
| 15.需要揉鼻／眼 | 0 | 1 | 2 | 3 | 4 | 5 | 6 |
| 16.需要反复的擤鼻涕 | 0 | 1 | 2 | 3 | 4 | 5 | 6 |

**鼻部症状**

在过去的7天里,您在多大程度上被下列问题所困扰?

|  | 没有困扰 | 几乎没有困扰 | 有些困扰 | 中等程度困扰 | 十分困扰 | 很困扰 | 极度困扰 |
| --- | --- | --- | --- | --- | --- | --- | --- |
| 17.鼻不通气 | 0 | 1 | 2 | 3 | 4 | 5 | 6 |
| 18.流鼻水 | 0 | 1 | 2 | 3 | 4 | 5 | 6 |
| 19.打喷嚏 | 0 | 1 | 2 | 3 | 4 | 5 | 6 |
| 20.鼻痒 | 0 | 1 | 2 | 3 | 4 | 5 | 6 |

**眼部症状**

在过去的7天里,您在多大程度上被下列问题所困扰?

|  | 没有困扰 | 几乎没有困扰 | 有些困扰 | 中等程度困扰 | 十分困扰 | 很困扰 | 极度困扰 |
| --- | --- | --- | --- | --- | --- | --- | --- |
| 21.眼痒 | 0 | 1 | 2 | 3 | 4 | 5 | 6 |
| 22.流泪 | 0 | 1 | 2 | 3 | 4 | 5 | 6 |
| 23.眼瞳 | 0 | 1 | 2 | 3 | 4 | 5 | 6 |
| 24.眼肿 | 0 | 1 | 2 | 3 | 4 | 5 | 6 |

**情感**

在过去的7天里,您有多少时间由于您的鼻／眼症状而被以下情感问题所困扰?

|  | 无 | 几乎没有时间 | 少部分时间 | 有些时间 | 大部分时间 | 绝大部分时间 | 所有时间 |
| --- | --- | --- | --- | --- | --- | --- | --- |
| 25.沮丧 | 0 | 1 | 2 | 3 | 4 | 5 | 6 |
| 26.内心不耐烦或不安宁 | 0 | 1 | 2 | 3 | 4 | 5 | 6 |
| 27.易恼怒 | 0 | 1 | 2 | 3 | 4 | 5 | 6 |
| 28.因症状而感到难堪 | 0 | 1 | 2 | 3 | 4 | 5 | 6 |
