# Supplementary material for: Effectiveness of strengthened stimulation during acupuncture for the treatment of allergic rhinitis: study protocol for a randomized controlled trial
Source: Trials. 2014 Jul 24;15:301. doi: 10.1186/1745-6215-15-301 (PMC4133069; doi:10.1186/1745-6215-15-301)
Supplement: Supplementary file 2 — Additional file 2: C-MMASS. Questionnaire to objectively scoring the sensation of de qi. (DOC 139 KB) [file 13063_2013_2188_MOESM2_ESM.doc]

没有感觉

轻微的

中等的

中等的

强烈的

不能忍受的

0

1

2

3

4

5

6

7

8

9

10

没有感觉

轻微的

中等的

中等的

强烈的

不能忍受的

0

1

2

3

4

5

6

7

8

9

10

没有感觉

轻微的

中等的

中等的

强烈的

不能忍受的

0

1

2

3

4

5

6

7

8

9

10

没有感觉

轻微的

中等的

中等的

强烈的

不能忍受的

0

1

2

3

4

5

6

7

8

9

10

没有感觉

轻微的

中等的

中等的

强烈的

不能忍受的

0

1

2

3

4

5

6

7

8

9

10

没有感觉

轻微的

中等的

中等的

强烈的

不能忍受的

0

1

2

3

4

5

6

7

8

9

10

没有感觉

轻微的

中等的

中等的

强烈的

不能忍受的

0

1

2

3

4

5

6

7

8

9

10

没有感觉

轻微的

中等的

中等的

强烈的

不能忍受的

0

1

2

3

4

5

6

7

8

9

10

没有感觉

轻微的

中等的

中等的

强烈的

不能忍受的

0

1

2

3

4

5

6

7

8

9

10

没有感觉

轻微的

中等的

中等的

强烈的

不能忍受的

0

1

2

3

4

5

6

7

8

9

10

酸痛

疼痛

压迫感

沉重感

针刺感

麻痹感

渊感

胀痛感

温暖感

冻感

没有感觉

轻微的

中等的

中等的

强烈的

不能忍受的

0

1

2

3

4

5

6

7

8

9

10

没有感觉

轻微的

中等的

中等的

强烈的

不能忍受的

0

1

2

3

4

5

6

7

8

9

10

跳动感

其他
